# Optimizing Thermal Pressing of Airlaids with Machine Learning

**DOI:** 10.1021/acsomega.6c02120

**Published:** 2026-05-20

**Authors:** Hannu Rummukainen, Tuomo Hjelt, Mikko Mäkelä

**Affiliations:** 3259 VTT Technical Research Centre of Finland Ltd., PO Box 1000, 02044 VTT Espoo, Finland

## Abstract

Airlaying is a promising alternative to conventional papermaking that does not require extensive drying and can potentially decrease the energy consumption of the forest industry. The key limitation of airlaids is weak fiber bonding, which results in low strength. Strength can be improved with thermal pressing, which involves multiple process parameters whose relationships with strength are not known. Here, we addressed this problem by combining deterministic linear models and probabilistic machine learning to improve airlaid properties by optimizing the conditions in thermal pressing. Our approach starts with a fractional factorial design as the initial sampling strategy to quantify the independent and interpretable variable effects and their interactions. We show how these resource-efficient designs can be easily complemented with a few additional experiments to identify more complicated behavior using a formal statistical test. We then identified three main challenges in optimizing the pressing conditions for our airlaids and tackled them with Bayesian optimization. Bayesian optimization improved the mechanical and physical properties of our airlaids, which showed tensile performance comparable to or 10% higher than traditional wet-laid paper, although bulk was still 30% lower than wet-laid paper. Our results are important, as they suggest that the middle layer of cardboard could potentially be replaced with a thermally pressed airlaid.

## Graphical abstract

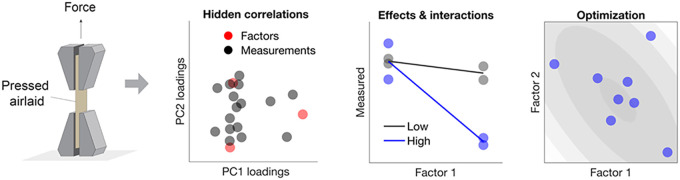

## 1 Introduction

Environmental concerns and the need to reduce fossil fuel dependency have accelerated the development of energy-efficient processes. Energy efficiency is particularly important for the pulp and paper industry, which is the third-largest energy consumer within the European Union.^1^ Conventional papermaking relies on dispersing fibers and additives in water, which must be removed by drying. The drying of a wet fiber web consumes approximately 50% of the total energy required for papermaking.^2^ Airlaids are alternative cellulosic materials that are produced from a web of dry pulp fibers and were originally developed to omit the drying step. A key limitation is their poor web bonding ability, which results in low strength. Strength is a critical property for paper products, particularly in packaging applications, and mechanical, chemical, or thermal consolidation methods are typically used to improve airlaid strength.^3^ Improving the bonding and strength of airlaids is essential for their adoption as a viable alternative to conventional papermaking.

Web strength has been enhanced by thermally pressing pulps with high lignin content.^4,5^ Thermal pressing, however, involves multiple controllable process parameters—such as pressure, pressing time, temperature, and prepress moisture content—and their precise relationships with web strength are not known. We tackled this challenge by combining deterministic linear models with probabilistic machine learning to improve the properties of pressed airlaids. Our approach started with a fractional factorial design^6^ as the initial sampling strategy to quantify independent and interpretable variable effects and their interactions. These resource-efficient designs were developed for variable screening and can be easily complemented with additional experiments to identify more complicated behavior using a formal statistical test. We illustrate how the results from this initial sampling strategy were interpreted with principal components analysis^7^ and linear regression models.^8^ Principal components provide a useful overview of the underlying correlations across many experimental variables and material features in just a couple of interpretable dimensions, and individual regression models enable a more detailed evaluation of specific material features. We then leveraged the available experiments as initial information for Bayesian optimization.

Bayesian optimization is an appealing machine learning alternative to traditional design of experiments in complex optimization tasks.^9–13^ The principles for optimization in design of experiments were originally formulated by a chemist and a statistician in the 1950s^14^ and are still considered as the gold standard in industrial experimentation.^15^ These traditional methods minimize prediction errors over the entire design range using a predetermined sampling plan,^16^ which does not allow using new information to improve the sampling strategy.^10^ Bayesian optimization combines a nonparametric surrogate model with an acquisition function to iteratively sample the design space. Modeling both the unknown response and its uncertainty enables choosing between *exploration and exploitation*, i.e., whether to reduce model uncertainty by experiments far away from previous experiments in the design space, or to seek improvement near the most promising experiments. Bayesian optimization algorithms converge to globally optimal values under weak theoretical assumptions and have been found to be sample-efficient in many practical applications.^17^ We hypothesized that combining deterministic linear models with Bayesian optimization would enable us to compare and interpret the effects of pressing conditions on airlaid properties and to find optimal conditions to generate comparable properties to those of traditional wet-laid paper.

## 2 Materials and Methods

### 2.1 Pressing Experiments and Material Features

We used chemi-thermomechanical pulp with a freeness number of 600 (Rottneros AB, Sunne, Sweden) as the pulp raw material. The dry pulp was first disintegrated with a laboratory-scale hammermill. The airlaid structures were prepared with a laboratory-scale Walkisoft (Anpap) drum former machine. The moisture content of the airlaids was adjusted by spraying water on the airlaid sheets, and the moistened sheets were stored in sealed plastic bags for a minimum of 4 h. The final moisture content of the airlaids was varied in the range 0–20% of the weight of the airlaid samples. The airlaids were pressed with a Scientific hot-pressing machine (Labtech Engineering Co., Ltd.) by heating both the upper and lower plates of the press. The press temperatures were controlled between room temperature (approximately 24 °C) and 180 °C. The pressing pressures and times were adjusted to the range 10–50 kg cm^–2^ and 1–10 min, respectively.

The properties of the pressed airlaids were determined by using standard methods in the pulp and paper field. The tensile index was determined by straining the pressed airlaids to break at a constant rate of elongation. The tensile testing was done with a Lloyd LS5 device (Lloyd Instruments) according to the ISO 1924-3 standard by recording tensile force and elongation using a 100 N load cell. Pressed airlaid bulk was determined as the ratio of airlaid thickness to its basis weight in cubic centimeters per gram (cm^3^ g^–1^) following the standard ISO 12625-3:2014. Basis weight measured the weight of an airlaid per unit area and was expressed in grams per square meter (g m^–2^).

### 2.2 Initial Sampling Strategy

We identified five potentially important experimental variables for controlling the pressing conditions. These quantitative variables were pressing pressure (kg cm^–3^), pressing time (min), pressing temperature (°C), airlaid moisture content (%), and airlaid composition. Airlaid composition was controlled by adjusting the share of chemi-thermomechanical pulp and fluff pulp in the binary fiber blend. Fluff pulp is a soft, absorbent, cellulosic fiber produced from spruce and pine. We used the controlled variables to determine a 2^5–1^ fractional factorial design, which was an orthogonal half-fraction of the 2^5^ full factorial design on five variables. The full factorial design, the defining relation, and the final fractional factorial design are illustrated in Tables S1 and S2 in the Supporting Information. We added three additional center-point experiments to evaluate repeatability and to test for potential curvature in the main effects.

### 2.3 Linear Models

We determined the correlations across the controlled variables and the determined material features with principal components analysis. The experimental data were compiled into a 19 × 11 matrix, where individual experiments were given as rows and the variables and features as the corresponding columns. The columns were normalized to unit variance and zero mean to compare variables given in different units. The data was decomposed according to the general principal components model, eq 1

$$Z_{m}=\overset{n}{\underset{i=1}{\sum }}t_{i}p_{i}^{T}+E_{n}$$ 1

where ***Z_m_*** denoted the normalized and mean-centered data matrix, ***t_i_*** the principal component scores, ***p_i_*** the corresponding loadings, and ***E**_n_* a matrix of model residuals after *n* components. The loadings of the first principal component were determined by maximizing the variation in the corresponding scores,^7^ and all the subsequent loadings were determined to be orthogonal to the previous ones.

Individual linear regression models were then built for chosen material features. We started with an initial model equation that included the main effects and the two-variable interactions, eq 2

2 $$y=\beta_{o}+\sum_{i=1}^{5}\beta_{i}x_{i}+\sum_{i<j=2}^{5}\sum \beta_{\mathrm{ij}}x_{i}x_{j}+e$$

where *y* denotes the determined material features, β denotes the coefficients for the average value, main effects, or two-variable interactions, *x* denotes the experimental variable values coded to [−1, 1], and *e* denotes the model residuals. A vector of model coefficients was determined using the least-squares estimate,^8^ and statistically insignificant (*p* < 0.10) model terms were excluded by backward elimination based on the largest *p*-values. These *p*-values were determined by comparing the coefficients against zero using a Student’s *t* test on the residual degrees of freedom.^8^

Prediction errors were estimated with leave-one-out cross-validation,^8^ and the residuals were determined according to eq 3

3 $${\mathrm{SS}}_{\mathrm{cv}}=\sum_{i=1}^{n}{\left(\frac{e_{i}}{1-h_{\mathrm{ii}}}\right)}^{2}$$

where SS_cv_ denotes the sum of squares of the cross-validation errors, *e*_*i*_ denotes a model residual, and *h*_*ii*_ denotes the *i*th diagonal element of the hat matrix **H = X­(X^T^X)^−1^X^T^**, where **X** refers to a matrix of coded variable values in the model, including a column of ones for the mean term. Potential curvature was evaluated based on the normalized difference in the means of the fractional factorial and center-point experiments,^18^ eq 4

4 $${\mathrm{SS}}_{c}=\frac{n_{F}n_{C}{\left({\mathrm{y̅}}_{F}-{\mathrm{y̅}}_{C}\right)}^{2}}{n_{F}+n_{C}}$$

where SS_c_ denotes the sum of squares for quadratic curvature, *n* denotes the number of the fractional factorial (*n*_F_) or the center-point experiments (*n*_C_), and *y̅*_F_ and *y̅*_C_ denote the corresponding feature means, respectively. The sum of squares for curvature from eq 4 was compared against the sum of squares of the model residuals using an *F* test. The *F* test was determined on 1 and *k* – 1 degrees of freedom, where *k* refers to the degrees of freedom of the residuals, with the null hypothesis that all quadratic terms would equal zero and there was no significant curvature to model with a more complicated model. The deterministic linear models were determined using in-house code programmed in MATLAB (The MathWorks, Inc.).

### 2.4 Machine Learning

We optimized the pressing conditions for the airlaids with Bayesian optimization. We focused on maximizing the tensile index and bulk of the pressed airlaids and combined these features into a single objective function, which was used in the Bayesian optimization algorithm to iteratively select the next pressing conditions. The specific form of the objective function was determined based on the results of the initial sampling strategy. The Bayesian optimization algorithm was initialized with the values of the controlled variables in the initial fractional factorial design and the corresponding objective values. The controlled variables were encoded in the range [0, 1], and on each iteration, the objective values from all preceding experiments were normalized to zero mean and unit standard deviation. We used a Gaussian process surrogate model with an unknown constant mean μ_*y*_, squared exponential covariance, and Gaussian observation noise with unknown variance σ_*y*_^2^ and zero mean.^19^ The observed objective values **y** were thus subject to a normal prior, eq 5

5 $$y∼N\left(\mu_{y}1,K+\sigma_{y}^{2}I\right)$$

with the elements of the covariance matrix **K** given by the squared exponential kernel, eq 6

6 $$k\left(x,x^{\prime }\right)=\exp\left(-\sum_{i=1}^{m}\frac{{{(x}_{i}-x_{i}^{\prime })}^{2}}{2l_{i}^{2}}\right)$$

where *m* is the number of controlled variables, **x** and **x**′ are vectors of controlled variable values, and *l*_1_, ..., *l*_*m*_ are length scale parameters for automatic relevancy determination.^20^ The observation noise variance σ_*y*_^2^ had a truncated log-normal prior LN(−4,1) with a hard lower bound of 10^–4^. The kernel length scale parameters *l*_1_, ..., *l*_*m*_ each had a truncated log-normal prior $LN\left(\sqrt{2}+\log2,\sqrt{3}\right)$ with a hard lower bound of 0.025.^21^ The mean μ_*y*_ had a uniform improper prior. After each experiment, the hyperparameters μ_*y*_, σ_*y*_^2^, and *l*_1_, ..., *l*_*m*_ were estimated by maximizing the posterior likelihood of the observed objective values.^19,22^

The controlled variables for each successive experiment were then selected by maximizing a Monte Carlo estimate of the log-noisy expected improvement acquisition function.^23,24^ We set up and ran the optimization using the Bayesian optimization algorithm implementation in the Ax/Botorch software package.^25^ The results of the Gaussian process models were visualized by using a set of uniform grid points *G* with 30 values for each controlled variable, and 30^*m*^ points in total. Given a posterior predictive distribution at point *x* with mean μ­(*x*) and variance σ^2^(*x*), the upper confidence bound at percentile *p* was of the form *f*_UCB_(*x*) = μ­(*x*) + Φ^–1^(*p*)­σ^2^(*x*), where Φ^–1^ was the quantile function of the normal distribution. The upper confidence bound of the optimum objective value was computed as $\max_{x\in G}f_{\mathrm{UCB}}\left(x\right)$ .

The effects of the controlled variable *x*_*i*_ were defined under the assumption that the other controlled variables were random and uniformly distributed. Using a random sample from the posterior of the Gaussian process on the uniform grid *G*, the effect at a particular value of *x*_*i*_ was estimated as, eq 7

7 $$E\left[Y|x_{i}\right]\approx \frac{1}{\left|G_{i}\left(x_{i}\right)\right|}\sum_{g\in G_{i}\;\left(x_{i}\;\right)}y_{g}$$

where *y*_*g*_ denotes the objective value sampled from the posterior at grid point *g* and *G*_*i*_(*x*_*i*_) denotes the subset of grid points with coordinate *i* equal to *x*_*i*_.

## 3 Results and Discussion

We studied the effects of thermal pressing conditions and airlaid composition on the physical and mechanical properties of pressed airlaids. The evaporation of water from a wet fiber web consumes approximately 50% of the energy used in papermaking, and in a paper machine, generally varies in the range 3.6–6.2 GJ per ton of removed water.^28^ This energy requirement would be considerably reduced if comparable cellulosic structures could be produced by pressing airlaid structures. We designed 19 individual experiments based on a fractional 2^5–1^ factorial design to screen the effects of the pressing conditions on airlaid properties. We compiled the experimental conditions and determined material features into a dataset, which we first decomposed with principal component analysis to study the correlations across the controlled variables and the material features.

The compiled dataset is given in Table S2 in the Supporting Information, and the results of the principal component decomposition are shown in Figure 1. The first two principal components explained 65% of the variation in the data, which was considerably higher than that of the subsequent components (Figure 1a). The first principal component explained the effects of the pressing pressure and temperature on the material features. As shown in Figure 1b, pressure and temperature showed a positive correlation with pressed airlaid density, strength, and tensile index. Higher pressures and temperatures led to higher airlaid density, which increased the strength and tensile index of the material (Table S2). The correlation between strength and density (Pearson correlation 0.66, *p* < 0.01) is well-known in paper physics, and a lot of research has been published to overcome this relation, as reaching comparable strength with lower density would lower raw material and production costs.^29^ Higher airlaid density decreased thickness and airlaid bulk (Figure 1b), which is a material feature commonly determined by paper physicists as the inverse of airlaid density.^29^

**Figure 1:**
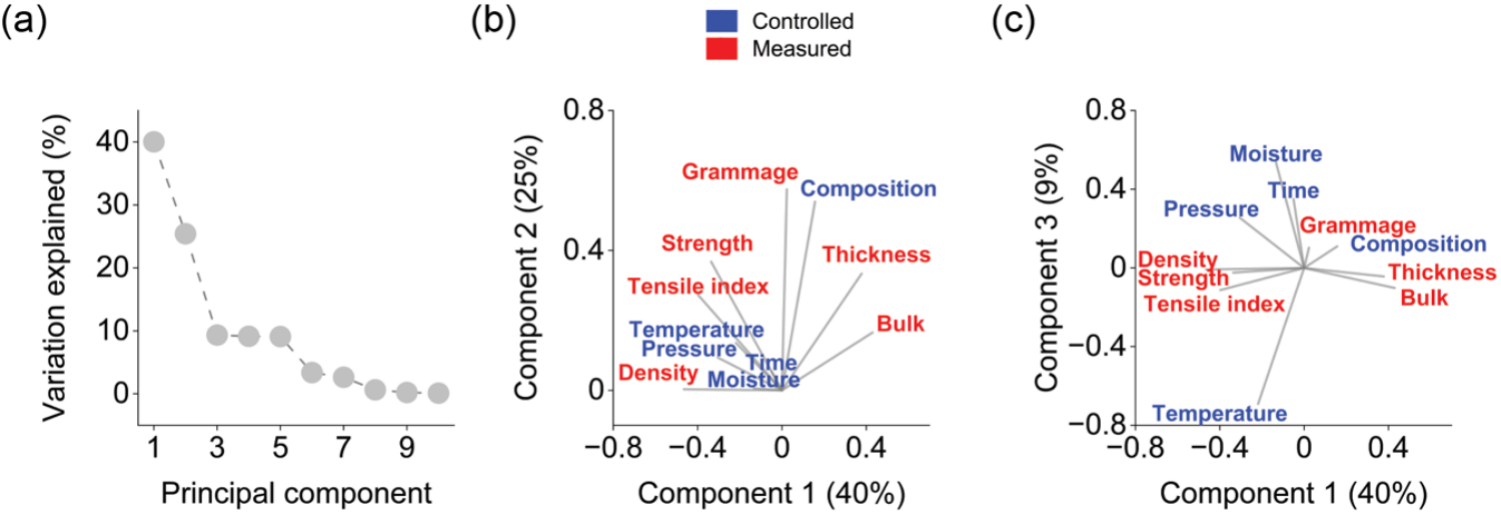
Principal components decomposition: variation explained by the principal components (a), the loadings of the first and second principal components (b), and the loadings of the first and third principal components (c).

The second principal component mainly explained the effects of airlaid composition on pressed airlaid properties. The share of chemi-thermomechanical pulp in the airlaid showed a positive correlation with airlaid grammage, strength, thickness, tensile index, and bulk (Figure 1b). These correlations were contrary to traditional papermaking, where fluff pulp generally shows higher bonding than other pulps due to higher flexibility and fines content,^30^ and indicated that lignin in the pulp plays an important role in strength improvement. Overall, the first two principal components provided a useful overview of the correlations across the controlled experimental conditions and the determined material features. The subsequent principal components mainly explained the remaining variation in the controlled design variables (Figure 1c).

**Figure 2:**
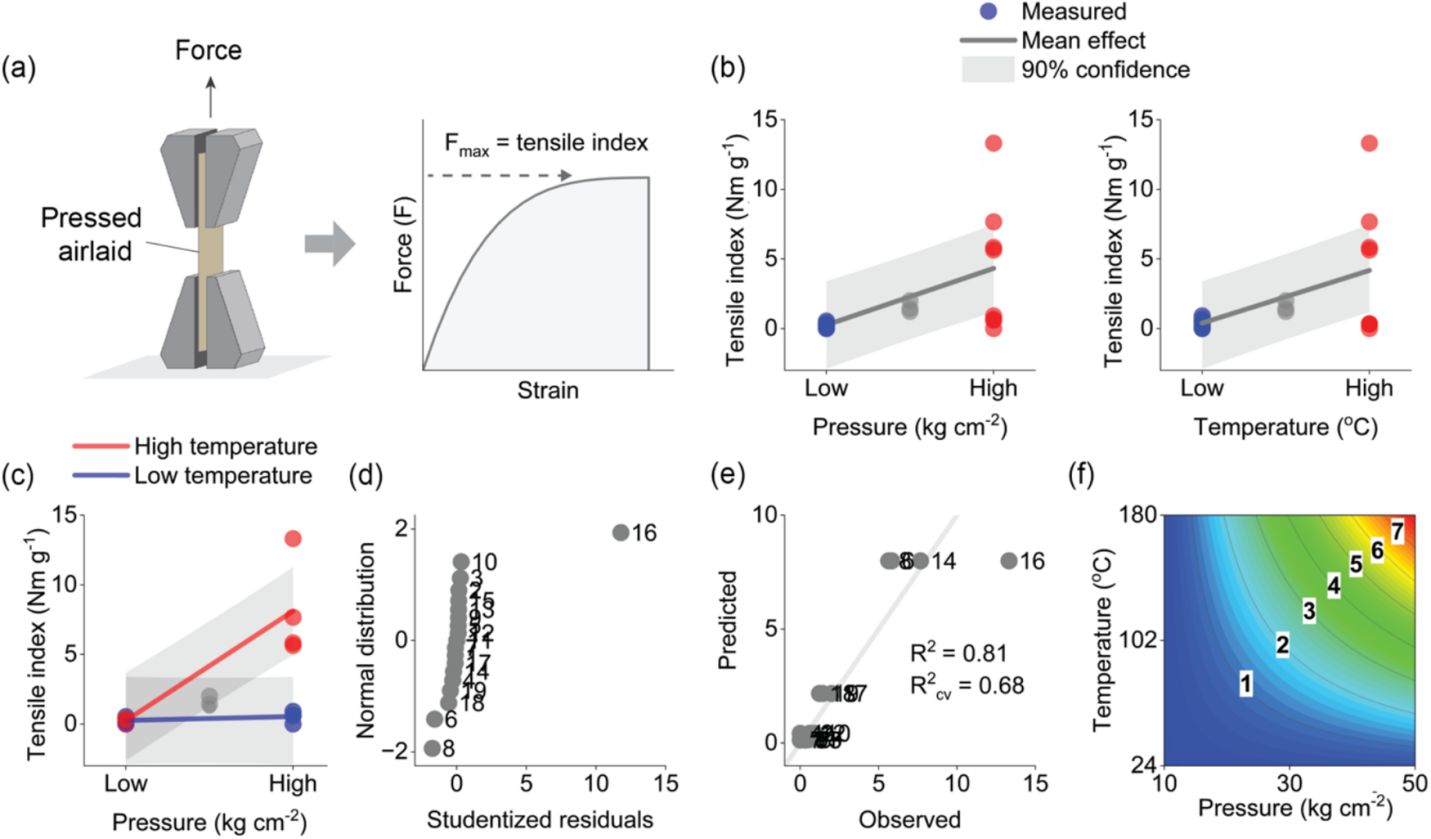
Linear tensile index model: the principle of the tensile index feature (a), the mean effects of pressure and temperature on tensile index (b), the interaction between pressure and temperature on tensile index (c), the studentized model residuals compared with the normal distribution (d), the predicted vs observed values (e), and a response surface of the predicted tensile index values as a function of pressure and temperature (f).

We then determined individual regression models for the chosen airlaid properties. The fractional factorial design enabled us to determine models with independent main effects of the controlled variables and all two-variable interactions, which were not confounded by the main effects or the other two-variable interactions. We focused specifically on tensile index and bulk, which we considered key indicators of the efficacy of the pressing procedure. The two regression models are summarized in Table 1. The models included 3–6 model terms in addition to the mean term and explained 81% of the variation in the determined material features. The determined *R*_cv_^2^ values indicated that the regression models explained 51–68% of the variation in the material features during leave-one-out cross-validation. We determined the normalized difference in the means of the fractional factorial and the center-point experiments and compared this variation against the variation in the model residuals using an *F* test. The results showed that airlaid bulk exhibited statistically significant quadratic curvature (*F*_1,11_ ratio of 5.47, *p* < 0.05). These observations suggested that a regression model with the main effects and their two-variable interactions was not sufficient to reliably estimate the changes in airlaid bulk based on the pressing conditions.

**Table 1:** A Summary of the Individual Regression Models for Specific Material Features

					Curvature	
Feature	Observations	Residual degrees of freedom	*R* ^2^	*R*_cv_^2^	*F*-ratio	*p*-value
Bulk (cm^3^ g^–1^)	19	12	0.81	0.52	5.59	0.04
Tensile index (N m g^–1^)	19	15	0.81	0.68	0.51	0.49

The principle of the tensile index measurement and the results of the linear regression model are shown in Figure 2. The results for airlaid bulk are given in Figures S1 and S2 for brevity. The tensile index feature was determined as the maximum force normalized with the basis weight of the airlaid sheet according to a standardized tensile test (Figure 2a). The subsequent model included the main effects for pressure and temperature and their two-variable interaction. Pressure and temperature showed positive model coefficients of 2.04 and 1.89 (standard errors 0.41, *p* < 0.01), respectively. The interaction between pressure and temperature (effect 1.90, standard error 0.41, *p* < 0.01) explained an additional 26% of the variation in the tensile index feature and provided valuable information on the effects of pressure and temperature. As shown in Figure 2c, a combination of high pressure and high temperature led to a larger improvement in the tensile index compared to either high pressure or high temperature alone. The tensile index of these airlaids increased to 13.3 N m g^–1^, which was higher than the rest of the pressed airlaids, as also indicated by the studentized model residuals (Table S2 and Figure 2d). We hypothesized that the combination of high pressure and high temperature likely softened the residual lignin in chemo-thermomechanical pulp, which improved bonding between the pulp fibers and increased airlaid strength.^31^ Thus, we did not exclude observation 16 from the sample set, which decreased the determined *R* ^2^ values of the model (Figure 2e). A response surface of the model predictions as a function of pressure and temperature is visualized in Figure 2f. The results showed that the tensile index increased toward higher pressures and temperatures, as indicated by the model coefficients.

We identified three main weaknesses in the traditional design of experiments for optimizing the pressing conditions for our airlaids. First, the coefficients in a multiple linear regression model are determined as a weighted sum of all the measurements, and the models are thus not particularly flexible at describing individual observations that are situated far away from the rest of the sample population (Figure 2d). An army of useful residual diagnostics has been developed to identify potential outlier observations to combat this weakness.^32^ Second, model-driven optimization in the design of experiments generally includes fitting and differentiating a quadratic regression function.^8^ Fitting such a function requires additional experiments after a fractional factorial design. We estimated that by narrowing down the design dimensions to four by only using chemi-thermomechanical pulp for producing the airlaids, we would have required at least eight additional experiments for determining a quadratic model and some validation experiments to test the potential optimum.^33^ Third, a quadratic regression function would have limited us to a specific set of model parameters. These parameters would likely not have been the best possible ones to describe the development of airlaid properties in the presence of additional chemical reactions, such as lignin plasticization.^31^

We replaced the optimization tools in the traditional design of experiments with Bayesian optimization to determine optimal pressing conditions for our airlaids. We targeted maximizing tensile index and bulk, but gave a higher importance to tensile index. We reviewed the tensile index and bulk results from the initial sampling strategy and defined the objective function *f* for Bayesian optimization as eq 9

9 $$f=\log_{10}\max\left(T,0.1\right)+{\frac{1}{5}\log}_{10}\max\left(B,0.1\right)$$

where *T* denotes the tensile index (N m g^–1^), *B* is the airlaid bulk (cm^3^ g^–1^), and the max function avoids numerical difficulties on small values of *T* and *B*. In the design range, *T* and *B* were both well above 0.1, and maximizing *f* was equivalent to maximizing *T* ^5^ *B*. Importantly, maximizing *T* ^5^ *B* was independent of the chosen units of tensile index and bulk, and in this form, maximizing bulk was equivalent to minimizing density. We chose these preferences to generate sufficient strength for the pressed airlaids while simultaneously minimizing pressed airlaid density. These properties could, in the future, enable us to use airlaid pressing as a simple and potentially energy-efficient methodology to produce novel lightweight packaging materials with minimal raw material costs.

We performed 10 iterative experiments using the pressing conditions suggested by the Bayesian optimization algorithm (Table S3). Our highest objective function value during initial sampling was reached by maximizing the values of the controlled variables, which generated a tensile index of 13.3 N m g^–1^ and a bulk of 2.2. cm^3^ g^–1^ for the pressed airlaids. As shown in Figure 3a, we found a higher objective value in one of the iterative experiments where reducing pressing temperature increased tensile index to 13.4 N m g^–1^ and bulk to 2.7 cm^3^ g^–1^. Several other promising pressing conditions were also discovered. The resulting tensile index values from these conditions were in the range of 9.4–11.9 N m g^–1^, with corresponding bulk values of 2.7–3.0 cm^3^ g^–1^. We compared these results with wet-laid sheets prepared from the same chemo-thermomechanical pulp fibers as our airlaid sheets.^34^ As shown in Figure 3a, these wet-laid sheets showed tensile index and bulk values in the range of 11.3–12.3 N m g^–1^ and 3.8–4.0 cm^3^ g^–1^, respectively. These results indicated that the tensile properties of our pressed airlaids were comparable to or even higher than those of traditional wet-laid paper.

**Figure 3:**
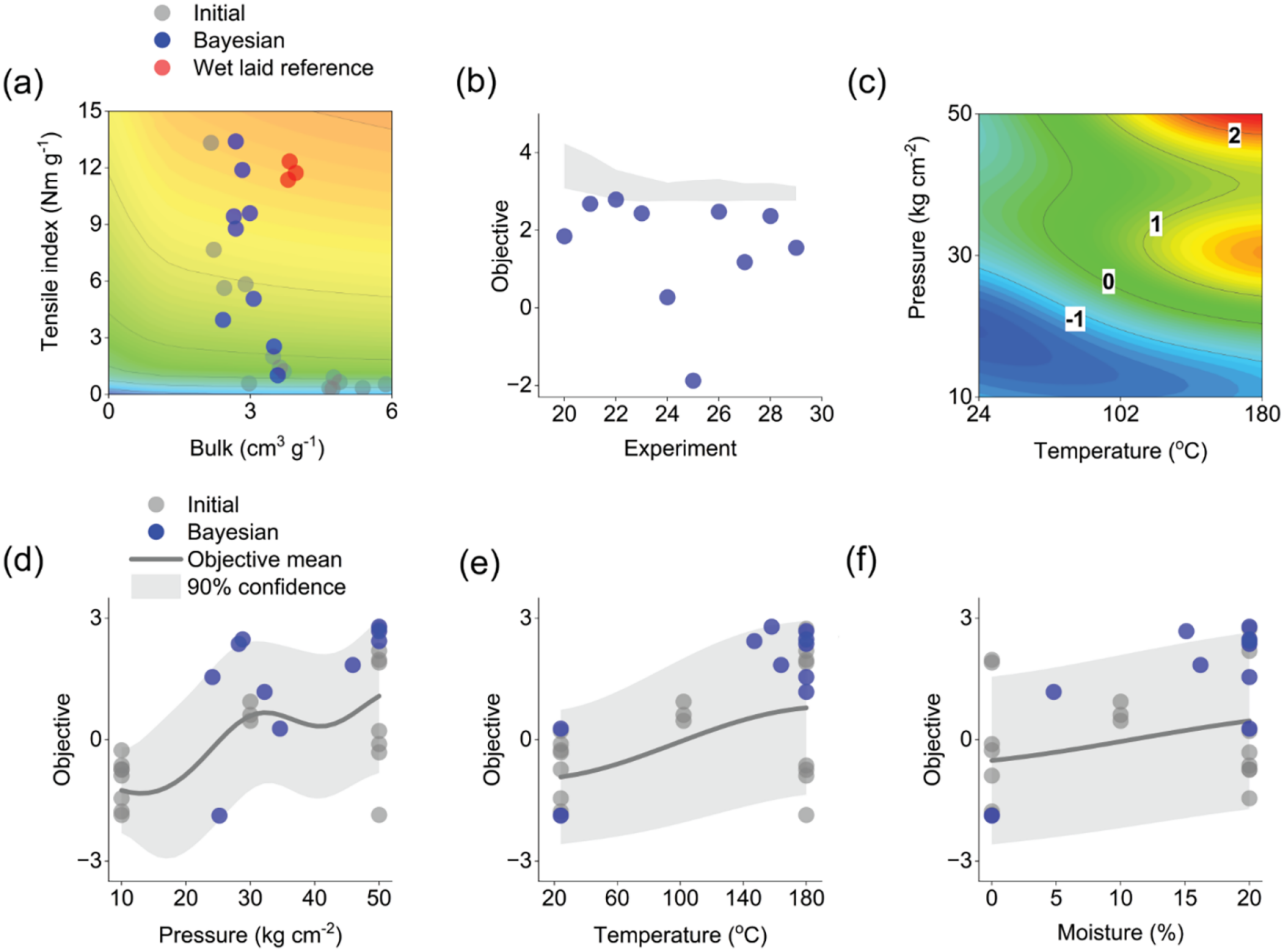
Measured tensile index and bulk values during the experiments with contours of the objective function used in Bayesian optimization (a), observed objective function value and estimated 0–95% percentile range of the optimum on each iteration (b), a response surface of the Bayesian optimization objective function mean as a function of temperature and pressure with an airlaid moisture content of 10% and a pressing time of 5.5 min (c), effects of the controlled variables pressure, temperature and airlaid moisture content on the objective value based on their conditional expectation and the corresponding 5–95% percentile range (d–f). Experiments resulting in low tensile index and high bulk values are not shown in part (a), see Table S2.

Overall, comparable tensile index properties were achieved with a lower airlaid bulk by using Bayesian optimization. We also substantially reduced the modeled uncertainty in the highest objective function value in the design space (Figure 3b). The upper confidence bound of the highest observed objective function value, 2.7, was reduced to 3.1 at the 95% percentile after 10 iterations. The respective upper confidence bound at the 75% percentile was 2.9, which indicated that substantial improvements were unlikely to be found within the design space.

A response surface of the final objective function after 29 experiments is shown in Figure 3c. The highest objective function values were achieved in two distinct regions using pressing temperatures higher than 140 °C combined with medium or high pressing pressures (Figure 3c). The main effects of the four controlled variables on the objective function are visualized in Figure 3d–f by plotting the conditional expectation $E\left[y|x_{i}\right]$ of the final Gaussian process model as a function of *x*_*i*_. The 5–95% prediction intervals were derived from the conditional variance by assuming that the other controlled variables were random variables with a uniform distribution.^35,36^ Only the effect of pressure on the objective was clearly nonlinear at this level (Figure 3d). Overall, the optimization procedure enabled us to achieve comparable tensile index properties to those during the initial experiments with higher airlaid bulk and lower density.

## 4 Conclusions

We combined deterministic linear models and probabilistic machine learning to optimize the properties of thermally pressed airlaids. We used a fractional factorial design as an initial sampling strategy and determined linear models to interpret the effects of pressing conditions and their interactions on the airlaid properties. The statistical tests for curvature indicated that a linear interaction model was not sufficient to reliably estimate the changes in the airlaid bulk. We identified three main weaknesses in traditional design of experiments for optimizing the pressing conditions and replaced these tools with Bayesian optimization. Bayesian optimization improved airlaid properties after the initial sampling and identified several promising conditions for airlaid pressing. As a result, our airlaids showed tensile performance comparable to or 10% higher than traditional wet-laid paper, although bulk was still 30% lower than wet-laid paper. The conditional expectation of the final Gaussian process model enabled us to evaluate the effects of the pressing conditions on the objective and indicated that the effect of pressure on maximizing tensile index and bulk was clearly nonlinear. Overall, our results are important, as they suggest that the middle layer of cardboard could potentially be replaced with a thermally pressed airlaid.

## Supplementary Material



## Data Availability

The data are available in the manuscript Supporting Information; the MATLAB and Python codes are available in a computational notebook published in Zenodo (10.5281/zenodo.15102769).

## Notes

The full 2^5^ factorial design, the defining relation, and the final fractional factorial design (Tables S1 and S2); experimental results (Tables S2 and S3); and the determined effects, model residuals, observed vs predicted values, and the predicted response contour for the airlaid bulk model (Figures S1 and S2) (PDF)
