# Supplementary material for: Optimizing Thermal Pressing of Airlaids with Machine Learning
Source: ACS Omega. 2026 May 20;11(21):31552–8. doi: 10.1021/acsomega.6c02120 (PMC13234668; doi:10.1021/acsomega.6c02120)
Supplement: Supplementary file 1 [file ao6c02120_si_001.pdf]

Optimising thermal pressing of airlaids with machine learning

Hannu Rummukainen, Tuomo Hjelt, Mikko Mäkelä*

VTT Technical Research Centre of Finland Ltd., PO Box 1000, 02044 VTT Espoo, Finland

E-mail: mikko.makela@vtt.fi

Appendix

Table S.1: A 2^5 full factorial design on five variables A-E and the defining relation ABCDE to determine two orthogonal 2^{5-1} half-fractions. The minimum and maximum values of the variables were coded to [-1,1] according to common design of experiments methodology. The half-fraction used in the experiments is highlighted in gray.

Experiment	A	B	C	D	E	ABCDE
1	-1	-1	-1	-1	-1	-1
2	1	-1	-1	-1	-1	1
3	-1	1	-1	-1	-1	1
4	1	1	-1	-1	-1	-1
5	-1	-1	1	-1	-1	1
6	1	-1	1	-1	-1	-1
7	-1	1	1	-1	-1	-1
8	1	1	1	-1	-1	1
9	-1	-1	-1	1	-1	1
10	1	-1	-1	1	-1	-1
11	-1	1	-1	1	-1	-1
12	1	1	-1	1	-1	1
13	-1	-1	1	1	-1	-1
14	1	-1	1	1	-1	1
15	-1	1	1	1	-1	1
16	1	1	1	1	-1	-1
17	-1	-1	-1	-1	1	1
18	1	-1	-1	-1	1	-1
19	-1	1	-1	-1	1	-1
20	1	1	-1	-1	1	1
21	-1	-1	1	-1	1	-1
22	1	-1	1	-1	1	1
23	-1	1	1	-1	1	1
24	1	1	1	-1	1	-1
25	-1	-1	-1	1	1	-1
26	1	-1	-1	1	1	1
27	-1	1	-1	1	1	1
28	1	1	-1	1	1	-1
29	-1	-1	1	1	1	1
30	1	-1	1	1	1	-1
31	-1	1	1	1	1	-1
32	1	1	1	1	1	1

Appendix

Table S.2: The experimental design and the results from the initial sampling strategy. The rows have been reordered from Table A.1.

Index	Pressure (kg cm^{-2})	Time (min)	Temperature (°C)	Moisture (%)	Composition (% CTMP)	Grammage (g m^{-2})	Thickness (μm)	Density (kg m^{-3})	Bulk (cm^3 g^{-1})	Strength (N m^{-1})	Tensile index (Nm m^{-1})	Objective value
1	10	1	24	0	100	107.2	1534.6	69.9	14.3	0	0	-1.77
2	50	1	24	0	0	63.6	311	204.5	4.9	41.6	0.7	-0.11
3	10	10	24	0	0	54.4	319.5	170.9	5.9	29.4	0.5	-0.26
4	50	10	24	0	100	120.7	1124	107.3	9.3	0	0	-1.86
5	10	1	180	0	0	57.6	273.2	211	4.7	17.4	0.3	-0.89
6	50	1	180	0	100	138.2	400.4	345.1	2.9	806	5.8	1.98
7	10	10	180	0	100	118.3	1091	108.5	9.2	0	0	-1.86
8	50	10	180	0	0	55.5	135.7	409.8	2.4	313	5.6	1.91
9	10	1	24	20	0	55.6	298.4	186.8	5.4	19.1	0.3	-0.73
10	50	1	24	20	100	102.3	487.4	209.9	4.8	93.1	0.9	0.22
11	10	10	24	20	100	126.2	844.6	149.5	6.7	20.3	0.2	-1.45
12	50	10	24	20	0	58.2	173.2	337.6	3	34.3	0.6	-0.31
13	10	1	180	20	100	113	1089.2	103.8	9.6	38	0.3	-0.64
14	50	1	180	20	0	57	126.8	449.5	2.2	437	7.7	2.20
15	10	10	180	20	0	53.9	252	214	4.7	18.9	0.4	-0.74
16	50	10	180	20	100	163.3	353.6	461.9	2.2	2175	13.3	2.74
17	30	5.5	102	10	50	98.2	342	287.2	3.5	196.6	2	0.94
18	30	5.5	102	10	50	98.7	366	270.8	3.7	121.1	1.2	0.47
19	30	5.5	102	10	50	101.7	368	277.6	3.6	145.9	1.4	0.62

CTMP = chemi-thermo-mechanical pulp.

Appendix

Table S.3: The experiments selected by the Bayesian optimization algorithm and their results.

Index	Pressure (kg cm^{-2})	Time (min)	Temperature (ºC)	Moisture (%)	Composition (% CTMP)	Grammage (g m^{-2})	Thickness (mm)	Density (kg m^{-3})	Bulk (cm^3 g^{-1})	Strength (N m^{-1})	Tensile index (Nm m^{-1})	Objective value
20	45.9	6.90	164	16.2	100	98.8	303.1	326.0	3.07	501	5.07	1.85
21	50.0	6.71	180	15.1	100	102.1	289.2	353.0	2.83	1210	11.89	2.68
22	50.0	10.00	158	20.0	100	104.2	280.2	371.9	2.69	1390	13.40	2.79
23	50.0	5.87	147	20.0	100	100.3	265.5	378.1	2.65	945	9.43	2.44
24	34.6	1.00	24	20.0	100	106.3	378.8	280.7	3.58	109	1.02	0.27
25	25.2	10.00	24	0.0	100	101.3	870.7	116.4	8.60	0	0.00	-1.87
26	28.8	1.00	180	20.0	100	91.8	275.0	334.2	2.99	882	9.60	2.48
27	32.2	1.00	180	4.8	100	102.7	357.9	287.2	3.50	261	2.54	1.18
28	28.2	9.47	180	20.0	100	97.6	262.8	371.5	2.69	857	8.78	2.37
29	24.1	3.00	180	20.0	100	85.1	204.0	416.2	2.42	336	3.95	1.55

CTMP = chemi-thermo-mechanical pulp

Appendix

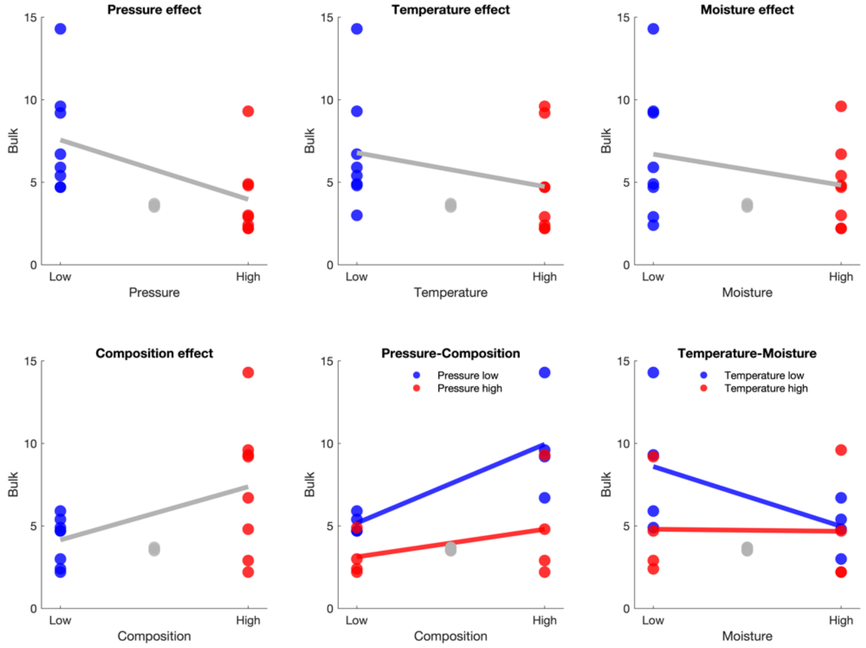

Fig. S.1: The effects of the controlled variables based on the individual regression model for airlaid bulk.

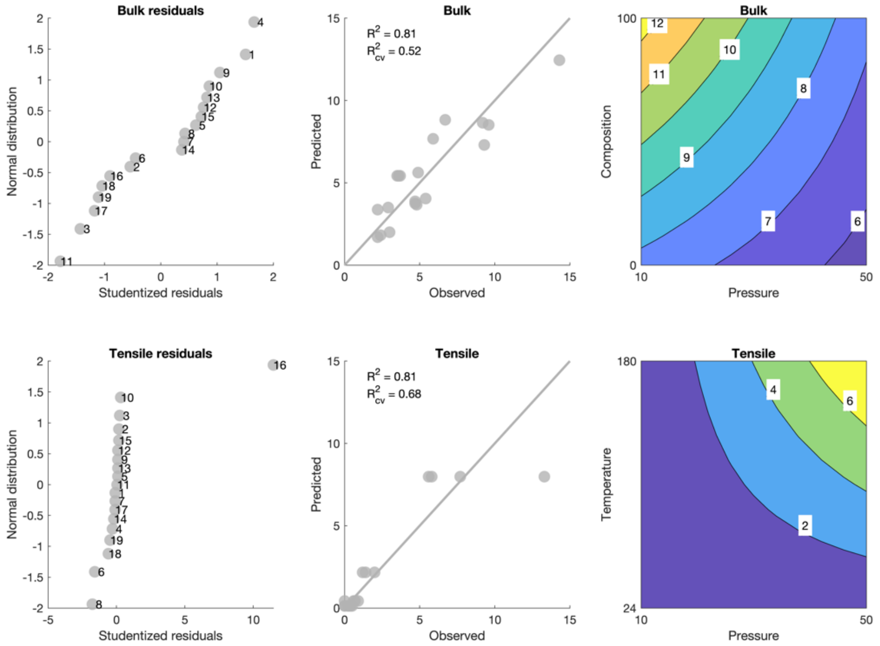

Fig. S.2: The Studentized residuals, predicted vs. observed values, and a response surface of the model predictions based on pressing pressure and airlaid composition for airlaid bulk (upper row).
